# Vaccines to Treat Substance Use Disorders: Current Status and Future Directions

**DOI:** 10.3390/pharmaceutics16010084

**Published:** 2024-01-08

**Authors:** Tangsheng Lu, Xue Li, Wei Zheng, Chenyan Kuang, Bingyi Wu, Xiaoxing Liu, Yanxue Xue, Jie Shi, Lin Lu, Ying Han

**Affiliations:** 1 National Institute on Drug Dependence and Beijing Key Laboratory of Drug Dependence Research, Peking University, Beijing 100191, China; lutsh@stu.pku.edu.cn (T.L.); lx@bjmu.edu.cn (X.L.); yanxuexue@bjmu.edu.cn (Y.X.); shijie@bjmu.edu.cn (J.S.); 2 School of Basic Medical Sciences, Peking University Health Science Center, Beijing 100191, China; 3 Peking-Tsinghua Centre for Life Sciences and PKU-IDG/McGovern Institute for Brain Research, Peking University, Beijing 100871, China; zhengw@stu.pku.edu.cn; 4 College of Forensic Medicine, Hebei Key Laboratory of Forensic Medicine, Collaborative Innovation Center of Forensic Medical Molecular Identification, Hebei Medical University, Shijiazhuang 050017, China; kcy58264@163.com; 5 Henan Key Laboratory of Neurorestoratology, The First Affiliated Hospital of Xinxiang Medical University, Xinxiang 453100, China; k121469895@163.com; 6 Peking University Sixth Hospital, Peking University Institute of Mental Health, NHC Key Laboratory of Mental Health (Peking University), National Clinical Research Center for Mental Disorders (Peking University Sixth Hospital), Beijing 100191, China; liuxiaoxing@pku.edu.cn

**Keywords:** vaccine, substance use disorders, addiction, immunotherapy, antibody

## Abstract

Addiction, particularly in relation to psychostimulants and opioids, persists as a global health crisis with profound social and economic ramifications. Traditional interventions, including medications and behavioral therapies, often encounter limited success due to the chronic and relapsing nature of addictive disorders. Consequently, there is significant interest in the development of innovative therapeutics to counteract the effects of abused substances. In recent years, vaccines have emerged as a novel and promising strategy to tackle addiction. Anti-drug vaccines are designed to stimulate the immune system to produce antibodies that bind to addictive compounds, such as nicotine, cocaine, morphine, methamphetamine, and heroin. These antibodies effectively neutralize the target molecules, preventing them from reaching the brain and eliciting their rewarding effects. By obstructing the rewarding sensations associated with substance use, vaccines aim to reduce cravings and the motivation to engage in drug use. Although anti-drug vaccines hold significant potential, challenges remain in their development and implementation. The reversibility of vaccination and the potential for combining vaccines with other addiction treatments offer promise for improving addiction outcomes. This review provides an overview of anti-drug vaccines, their mechanisms of action, and their potential impact on treatment for substance use disorders. Furthermore, this review summarizes recent advancements in vaccine development for each specific drug, offering insights for the development of more effective and personalized treatments capable of addressing the distinct challenges posed by various abused substances.

## 1. Introduction

Substance use disorder (SUD), a complex and pervasive public health issue, continues to exert a significant toll on individuals, families, and societies worldwide. The addictive properties of substances make quitting a formidable challenge for those affected. In recent years, many regions have witnessed a surge in drug overdose deaths, driven largely by the opioid crisis [1,2,3]. Overdoses not only result in tragic loss of life but also place additional stress on emergency medical services and first responders. Traditional treatment approaches, such as behavioral therapy and medication-assisted therapy (methadone, buprenorphine, naltrexone, naloxone), have made strides in addressing addiction [4]. Despite the fact that these medications can be used to treat drug addiction, their application is not without limitations. Behavioral therapy is coupled with a significant rate of treatment dropout and a high rate of relapse. Notably, antagonists such as naloxone and naltrexone are not entirely inactive in the absence of external opiates and interfere with the body’s endogenous opioid system, causing negative emotional effects in the long-term treated patient [5]. These issues can hinder the effectiveness of behavioral interventions. Therefore, there remains an unmet need for innovative and more effective interventions to address the challenges of addiction treatment. Ongoing research is exploring novel approaches, including the development of advanced medications, immunotherapies, and personalized treatment plans tailored to individual needs and circumstances [4]. Ultimately, the objective is to enhance the efficacy of addiction treatment and facilitate sustained recovery for individuals grappling with SUDs.

In recent years, the development of anti-drug vaccines has emerged as a promising frontier in the battle against SUDs. These vaccines, rooted in the principles of immunotherapy, offer a novel approach to addiction treatment. Unlike traditional pharmacological interventions, addiction vaccines harness the power of the immune system to combat addiction at its core [6]. Well-established research over the past six decades indicates that haptens, small molecules such as cocaine, nicotine, and heroin, typically do not elicit an immune response in isolation unless they are chemically bonded to immunogenic proteins. Therefore, to produce an anti-drug vaccine, the hapten is covalently linked to an immunogenic carrier protein, permitting the immune system to recognize the addictive substance or its metabolic products. This conjugation process enables the production of antibodies via B cells and initiates the activation of T cells, which are essential for generating robust antibody responses. A successful anti-drug vaccine can prevent the target addictive substance from entering the brain and neutralize drug doses. This immunization approach is designed to eliminate the rewarding and euphoric effects or alleviate cravings associated with drug use, thereby decreasing the motivation to relapse and continue drug consumption. However, it is important to consider that this strategy might not prevent drug users from consuming larger doses in an attempt to achieve the desired reward effects [7]. In addition, the side effects of anti-drug vaccines are minimal compared to some traditional pharmacological treatments, because antibodies produced by vaccines do not directly interact with drug receptors in the brain or other body tissues. Promising preclinical trial results regarding anti-drug vaccines provide optimism for ongoing and upcoming clinical treatment of SUDs. Clinical therapeutic vaccines are variable in individual response to active immunization arising from specific vaccine components, vaccine dose and schedule of vaccination, individual and/or baseline differences in immune response, sex, age, genotype, and individual drug use patterns [8]. However, clinical advantages of anti-drug vaccines seem to outweigh their disadvantages, potentially leading to enhanced treatment options in the field of addiction treatment [9,10]. In this review, we will delve into the intriguing domain of anti-drug vaccines, elucidating their mechanisms, current developmental status, and their potential to revolutionize addiction treatment. Although still in the experimental phase and facing formidable challenges, addiction vaccines offer a beacon of hope for individuals and communities grappling with addiction’s profound impact. See Table 1 for a summary of discussed anti-drug vaccines in this review.

## 2. The Mechanisms of Immunotherapies against SUDs

**Figure 1:**
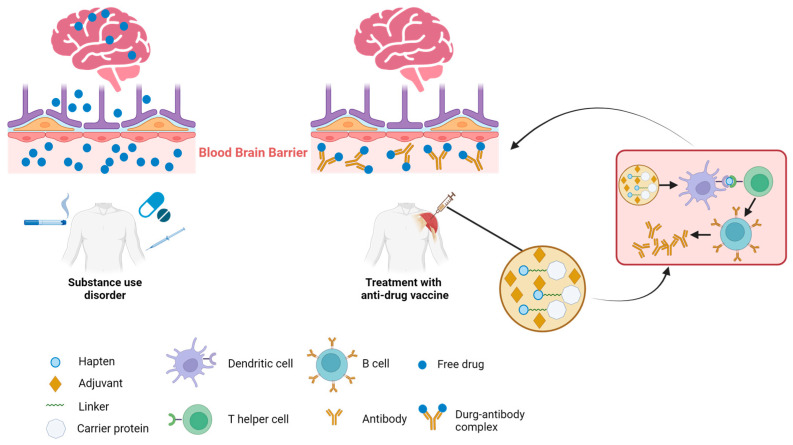
Mechanism of action of anti-drug immunotherapies. Multiple routes of addictive substance uptake into the body leading to drug transmission across the blood–brain barrier and subsequent interaction with brain (**left**). Intramuscular administration of a vaccine comprising the hapten conjugated to a carrier protein and adjuvants elicits an antibody response against the targeted drug. The presence of antibodies prevents the target drug from crossing the blood–brain barrier into the brain, thus mitigating the drug’s effects in the brain (**right**).

Potential detrimental effects of SUDs on various organ systems have been observed, with the most significant impact being on the central nervous system (CNS). Given the CNS’s inherent vulnerability to the adverse effects of drug abuse, immunotherapy emerges as a particularly effective therapeutic approach for addressing drug addiction, particularly when the CNS is involved. There are two key mechanisms underlying the effectiveness of immunotherapy for CNS-mediated drug addiction (Figure 1). The initial mechanism centers on the binding of drug molecules to antibodies, which are too large to pass through the blood–brain barrier (BBB) and penetrate into the brain. This action decreases the amount of free drug circulating and hampers the drug’s interaction with brain receptors, thereby mitigating its effects such as addiction, respiratory depression, and bradycardia. The secondary mechanism is characterized by decelerating the rate at which abused drugs reach the brain [32]. Antibody–drug binding has been shown to increase the half-life of the target drug. This is attributed to that the drug, once bound to the antibodies, becomes protected from enzymatic degradation. Consequently, this reduction in the drug’s clearance from the body results in an extended presence of the drug–antibody complex in circulation. This prolongs the period during which the drug is sequestered and prevented from interacting with its targets, such as receptors in the brain or other organs, thereby attenuating the pharmacodynamic effects of drugs [33]. Together, these mechanisms contribute to the vaccine’s ability to counteract ongoing drug overdose or diminish the reinforcing effects of the drug. By leveraging these mechanisms, immunotherapy holds a promise as a strategy to mitigate the impact of drug addiction, particularly when the CNS is significantly impacted. Further research is essential to refine and validate these mechanisms and their therapeutic potential.

## 3. Conjugate Vaccines

The addictive substances, such as heroin, oxycodone, morphine, nicotine, cocaine, and methamphetamine, exhibit a commonality in their small molecular structure, which precludes the induction of a robust immune response. To trigger an immune response, they need to be linked to an immunogenic carrier molecule. Indeed, in recent times, there has been a growing interest in conjugate vaccine development strategies that involve the design of specific haptens and their conjugation to immunogenic carrier molecules. This approach has garnered attention due to its versatility and potential to bring about structural modifications in a range of compounds that can target various drugs or even combinations of drugs [34,35,36]. The primary objective of these adjustments is to encourage the generation of potent antibody responses, a key element in the effectiveness of vaccination initiatives [37,38]. Conjugate vaccines have shown great promise as an immunotherapeutic intervention strategy for SUDs. In this approach, a hapten structurally similar to the target drug is linked to an immunogenic carrier protein. Conjugate vaccines act as immunoantagonists, meaning that they can counteract or mitigate the pharmacodynamics of the target drug. By eliciting the production of drug-specific antibodies, these vaccines prevent or reduce the drug’s pharmacological effects, providing a novel approach to managing substance abuse and addiction. Compared to conventional medication, immunization with conjugate vaccines presents several potential advantages for addressing SUDs. These benefits encompass the prospect of minimized side effects, convenient and cost-effective administration, and long half-lives of the generated antibodies that offer enduring protection against overdose by sequestering the abused drug in the periphery, thereby limiting its ability to cross the BBB. This enables treatment over the course of months, surpassing the metabolic and elimination rates of small-molecule therapeutics [13]. In addition, these antibodies demonstrate a significant degree of structural heterogeneity. An analysis indicated that both the sequence variations in the antigen-binding domain (Fab) and glycosylation at the CH2 domain (Fc) played simultaneous roles in the observed molecular microheterogeneity. These variations may have significant implications for the conformational stability and binding efficacy of the antibodies towards haptens [39]. In summary, this methodology facilitates the development of vaccines capable of eliciting an immune response specifically tailored to the target drug. Thus, in theory, conjugate vaccines can be custom-designed to target any drug or drug combination, rendering them a potentially invaluable strategy for countering substance abuse and related disorders.

## 4. The Key of Anti-Drug Vaccine Design

Anti-drug vaccines confront potential challenges, such as suboptimal hapten design and carrier molecule selection [39,40]. Furthermore, the preclinical development of these drug vaccines has frequently been inadequately stringent, as they have yet to consistently demonstrate the capacity to effectively antagonize various drug doses in diverse behavioral assays. The inability to effectively address and resolve these challenges has hindered the progression of drug vaccine research.

Immunogenicity is one of the most critical factors that govern the efficacy of a vaccine. Haptens are altered forms of the addictive substance capable of triggering an immune reaction. It is essential to design haptens that preserve the substance’s vital structural characteristics while also facilitating robust antibody attachment, a vital step in developing both antibody efficacy and specificity [41,42]. Haptens need to be structurally distinct from endogenous molecules to avoid cross-reactivity with normal cellular components. Haptens, small molecules representing the drug, alone may not stimulate a robust immune response. They need to be chemically linked to an immunogenic carrier molecule, typically a protein, to enhance their recognition by the immune system. Bovine serum albumin (BSA), diphtheria toxoid (DT), tetanus toxoid (TT), and keyhole limpet hemocyanin (KLH) have been widely investigated as protein carriers for anti-drug vaccine development [43,44]. These carriers help promote the activation of immune cells and the production of antibodies against the hapten [43]. The antibodies generated by the immune response should have high specificity and affinity for the addictive substance. This ensures effective binding and neutralization of the substance before it can reach its target receptors in the brain. The conjugate immunogens consisting of the hapten and carrier protein combination are paired with an adjuvant, which is a compound added to vaccines to enhance the immune response. Adjuvants stimulate immune cells and promote a stronger and longer-lasting antibody response. Selecting the right adjuvant is equally important for optimizing vaccine efficacy [45]. Certainly, all these essential factors must be optimized to achieve the most favorable outcomes in the development of anti-drug vaccines.

## 5. Recent Progress in Anti-Drug Vaccine Research

### 5.1. Opioid Vaccines

Prescription opioid (PO) pain relievers constitute the cornerstone of modern pain management therapy. However, they also rank among the most frequently misused and abused medications, such as oxycodone, hydrocodone, and especially fentanyl (the wave of overdose deaths attributed to illicit fentanyl since 2013 is unprecedented) [2,46]. Traditional pharmacological therapies, such as the mu-opioid receptor (MOR) agonists methadone and buprenorphine, have been useful in treating opioid dependence [47,48]. Additionally, MOR antagonists like naltrexone and naloxone are effective in reversing opioid overdoses [49]. However, these medications have not been fully successful in addressing the alarming increase in opioid abuse in recent times. Moreover, they can be associated with significant side effects, limiting their overall effectiveness and acceptance in some cases [50,51]. A vaccine-mediated pharmacokinetic strategy could combat the deadly and addictive effects of opioids. This approach involves using a small-molecule-immunogenic protein conjugate to trigger the production of drug-specific IgG antibodies. These antibodies can then attach to freely circulating opioid molecules, preventing them from entering the central nervous system (CNS) and inducing activation of the MOR [52]. The vaccination using an oxycodone hapten containing a tetraglycine linker at the C6 position conjugated to keyhole limpet hemocyanin (6OXY(Gly)4–KLH) has produced notable effects in relation to increasing drug binding, reducing drug distribution, and blunting analgesia for both oxycodone and hydrocodone [12]. The oxycodone vaccine (Oxy(Gly)4-sKLH) will be tested in participants with opioid use disorder (OUD). Its Phase I and II clinical trials will be completed this year (NCT04458545) [11]. Indeed, prior research has demonstrated that TT, native keyhole limpet hemocyanin (nKLH), and GMP-grade KLH dimer (dKLH) carriers provide consistent oxycodone derivatized at the C6 position (6OXY) conjugate vaccine immunogenicity and attenuate oxycodone-induced hotplate antinociception [53]. OXY-dKLH also effectively diminished oxycodone-induced respiratory depression and heart rate hold, which are significant implications in addressing the opioid overdose crisis [52]. The synthesis of oxycodone-TT (Oxy-TT) and hydrocodone-TT (Hydro-TT) vaccines was successfully accomplished. A recent study has conclusively demonstrated that Oxy-TT and Hydro-TT vaccines effectively reduce mortality resulting from lethal doses of hydrocodone and oxycodone [13]. This finding highlights the potential of active vaccination as a viable approach to combat the ongoing prescription opioid overdose epidemic.

Due to the fact that synthetic opioid-related (predominately illicit fentanyl and fentanyl analogs) overdose rose dramatically from 2014 to the present [54,55], the development of novel therapeutics is a public health priority. Indeed, fentanyl vaccines have shown promising effects in preclinical studies, particularly in providing protection against overdose and mitigating the analgesic and respiratory effects of fentanyl in rhesus monkeys [56,57]. In addition, a conjugate fentanyl-TT vaccine significantly blunted fentanyl reinforcement and prevented the expression of withdrawal-associated increases in rats [14]. Given the evolution in drug supply over time, including increased synthesis of fentanyl analogs, a successful vaccine must demonstrate efficacy for fentanyl and related compounds, which may complicate development. In a preclinical animal study, FEN-CRM + dmLT (a fentanyl-like hapten was combined with the carrier protein CRM197 and an adjuvant derived from heat-labile enterotoxins from *E. coli* (LT) named dmLT) generated significant amounts of anti-fentanyl antibodies that were associated with a significant blockade of fentanyl’s analgesic effects in mice. In the meantime, cross-reactivity assays demonstrated that anti-fentanyl antibodies exhibit binding affinity to fentanyl and sufentanil, while displaying no binding interaction with morphine, methadone, buprenorphine, or oxycodone [15]. In another study, Carfen-ester-TT (the methyl ester functional group of carfentanil as the point of linker attachment) and Carfen-p-phenyl-TT (positions the linker at the para position of the phenethyl ring) can effectively target fentanyl and carfentanil, eliciting high-affinity antibodies against both drugs in mice [58]. These results indicated a specific recognition and binding capacity of the antibodies to fentanyl-related compounds, supporting the targeted nature of the vaccine against these opioids. Recently, the incorporation of synthetic toll-like receptor (TLR) 7/8 agonists as vaccine adjuvants increased vaccine efficacy against fentanyl challenge, overdose, and self-administration in mice, rats, or Hanford miniature pigs [59,60]. The development of opioid immunotherapies poses significant challenges. However, as the opioid crisis continues to persist, the pressing need for novel treatment approaches in the face of the enduring fentanyl overdose crisis is undeniable. An anti-fentanyl vaccine emerges as a promising avenue, offering a potentially innovative treatment and risk-mitigation strategy for overdose. Importantly, this approach may have a broader applicability to a larger target audience when compared to currently available interventions.

Heroin is a potent and highly addictive opioid drug that poses significant risks to individuals and public health [61]. In the treatment of heroin abuse, long-acting oral opioid agents are often employed to provide a controlled and stable replacement therapy, such as methadone, levo-acetylmethadol (LAAM), and buprenorphine. These medications are used as part of medication-assisted treatment (MAT) approaches and work by mimicking drug action at the opioid receptors, which helps to alleviate cravings and withdrawal symptoms. Naltrexone and naloxone are other medications commonly used as pharmacotherapies for both abstinence and prevention of relapse in heroin addiction and OUD treatment. There are obvious deficiencies in both drug mimicry and drug antagonism. MAT may expose individuals to opiates, leading to tolerance and withdrawal from the treatment agent. Naltrexone and naloxone do not remain inactive in the absence of exogenous opioids. Instead, they actively block the binding of both exogenous opioids (e.g., heroin) and the body’s endogenous opioids (e.g., enkephalins and endorphins) to opioid receptors in the brain and other parts of the body causing negative emotional effects in the long-term treated patient. The first studies to provide the proof of concept for the use of active vaccination for treating heroin dependence were by Bonese et al. [62]. However, this approach was ultimately abandoned due to apprehensions that individuals might readily transition to other opioids. Heroin is a prodrug, which means that it undergoes chemical transformations in the body to form active metabolites. It rapidly undergoes deacetylation, converting it into two primary metabolites: 6-acetylmorphine (6 AM) and morphine. The early report of a 6 AM-like hapten known as morphine-6-hemisuccinyl-bovine serum albumin (M-6-H-BSA) was explored 50 years ago by Bonese et al. [62]. Subsequently, Anton et al. developed a morphine-tetanus toxoid (M-TT) vaccine, which has shown the capability to elicit a strong and long-lasting immune response against heroin [16]. A similar vaccine utilizing 6-succinylmorphine conjugated to BSA (M-6-S-BSA) could generate high-titer anti-morphine antibodies with satisfactory specificity [17]. Due to the fact that morphine is another component of the heroin metabolite, the utilization of a morphine-based hapten linked to KLH (M-KLH) has been shown to have the potential advantage of multivalent opioid vaccines. M-KLH could induce substantial levels and concentrations of antibodies with a strong affinity for heroin, 6-AM, and morphine and reduce brain 6-AM and morphine concentrations [18]. M-KLH could also effectively prevent the heroin-primed reinstatement and reduce heroin-induced locomotor activity [63,64]. Subsequently, a first-generation heroin vaccine based on two heroin- and morphine-like haptens exhibited blocked antinociceptive effects of heroin and inhibited acquisition of heroin self-administration [65]. KLH-6-SM (keyhole limpet hemocyanin-6-succinylmorphine) is regarded as a candidate vaccine for opioid dependence depending on its ability to produce antibodies that show specificity for morphine or other heroin metabolites [19]. A significant leap forward was made in a second generation heroin vaccine, which has been identified through optimization of the adjuvant (CpG ODN + alum), carrier protein (TT) and hapten (HerCOOH), achieved a greater than 15-fold heroin ED50 shift in rodents and mitigated heroin-induced decreases in operant responding in non-human primates [66].

To date, more and more conjugate vaccines designed for heroin have consistently shown strong antibody responses targeting heroin and its metabolites. These vaccines have resulted in decreased opioid concentrations within the brains of vaccinated animals, as well as reductions in heroin-induced motor and behavioral activity [10,67,68]. Regioselective deuteration of a heroin-hapten elicited both greater quantities as well as equivalent or higher-affinity antibodies towards heroin and 6-AM. This deuteration could blunt heroin analgesia in murine behavioral models [69]. However, vaccines developed for opioid use disorder face the distinct challenge of creating antibodies that target both the active metabolites and the parent compound. Additionally, these vaccines need to accommodate the practice of users switching between different opioids when their preferred drug is unavailable. For the first challenge, the majority of vaccines developed for heroin or morphine use morphine-derived haptens, because the resulting antibodies tend to exhibit a strong affinity for both morphine and its active metabolites. This broad binding capability enhances the vaccine’s effectiveness in targeting various opioids and their effects [18,19]. For the second, multivalent vaccines offer a promising solution to the issue of opioid users switching between different opioids. Rats that received the bivalent vaccine exhibited higher levels of antibodies against the individual immunogens compared to those receiving separate monovalent vaccines [70]. Up until now, no vaccines for heroin or morphine have received approval for human use. While promising results have been achieved in preclinical models, research endeavors are required to transition these above potential candidate vaccines into human clinical trials.

### 5.2. Nicotine Vaccines

Tobacco use disorder stands as the primary cause of both disease and preventable fatalities globally. Nicotine, a psychoactive ingredient naturally present in tobacco, functions as an agonist for nicotinic acetylcholine receptors and leads to escalation and compulsive-like pattern of smoking. Quitting smoking without medical interventions can be a challenging and often very difficult endeavor for smokers [71]. An effective approach for addressing tobacco use disorder involves diminishing the psychoactive impact of nicotine by impeding its access to the brain. Early preclinical studies suggest that immunization results in ~30–90% less nicotine entering the brain after acute nicotine exposure and slows nicotine elimination from the body, which may contribute to reduction in smoking [72]. 

In the early 21st century, some Phase II clinical trials have failed due to side effects (such as flu-like symptoms, injection site pain, headache, and so on) or inadequate antibody titers [22]. Nabi Biopharmaceuticals has reported encouraging outcomes from Phase II trials of NicVAX. The NicVAX vaccine exhibited a favorable safety profile and was well-tolerated by participants. Notably, the vaccine prompted the production of elevated levels of anti-nicotine antibodies [73]. A clinical study demonstrated that immunization using NicVAX led to a significant decrease in β2*-nicotinic acetylcholine receptor (β2*-nAChR) occupancy by nicotine. This effect was achieved by trapping nicotine in the bloodstream and subsequently reducing its passage into the brain [20]. This strategy could potentially facilitate a gradual reduction in dependence, resulting in decreased cravings and thwarting relapse prompted by nicotine re-exposure. However, Phase III trials demonstrated identical rates of abstinence between active and placebo vaccine conditions [74,75]. More efforts to achieve this pharmacokinetic goal through vaccines have been undertaken [76]. A novel nanoparticle (NP)-based nicotine vaccine (NanoNicVac) was developed [23]. Thereafter, various novel nicotine vaccines were developed according to the density and location of haptens [77,78]. The strategic integration of toll-like receptor (TLR) adjuvants increased total anti-nicotine IgG titers, affected IgG subtype distribution in mice, and reduced the brain nicotine levels in mice after nicotine challenge. Such design has the potential to amplify the immunological effectiveness of the hybrid nanoparticle-based nicotine vaccine [79]. It was observed that a nicotine vaccine containing 20% PEGylation (referred to as NanoNicVac 20.0) exhibited notably higher stability in comparison to vaccines with lower levels of PEGylation. The inclusion of 20% PEGylation in NanoNicVac appears to confer on the vaccine a favorable combination of enhanced safety, superior stability, and heightened immunological effectiveness in mice [80]. FH VLPs (FljB was displayed at high densities on hepatitis b core (HBc) virus-like particle (VLP) surface upon c/e1 loop insertion) were found to be a more immunogenic carrier than the widely used keyhole limpet hemocyanin for nicotine vaccine development with good local and systemic safety [24]. Recently, Hu lab developed two nicotine vaccines using lipid-poly (lactic-co-glycolic acid) (PLGA) and lipid-polylactic acid (PLA) hybrid nanoparticles, respectively. It was observed that the lipid-PLA-based nicotine vaccine (referred to as PLA vaccine) exhibited significantly enhanced stability compared to the lipid-PLGA-based nicotine vaccine (referred to as PLGA vaccine) in both in vitro and in vivo settings. However, mice immunized with either the PLA vaccine or the PLGA vaccine alone did not achieve sufficiently high concentrations of long-lasting nicotine-specific antibodies. Interestingly, mice that were initially administered two injections of the PLGA vaccine, followed by a subsequent third injection of the PLA vaccine, demonstrated elevated concentrations of nicotine-specific antibodies persisting for up to three months [81]. Intranasal immunization with a Nic-KLH/MPL vaccine can induce systemic and mucosal antibodies that specifically neutralize nicotine [82]. Therefore, for these attempts to be successful, antibody titers must be sufficient to effectively block nicotine’s entry into the brain. 

Another crucial point to consider is that many animal models used for vaccine studies entail the intravenous or subcutaneous administration of nicotine in isolation. This methodology significantly diverges from the real-world scenario of nicotine inhalation, tobacco consumption, and exposure to the multitude of chemicals found in cigarettes that humans experience. Indeed, antagonizing the effects of nicotine has shown promise in conjunction with varenicline, considered the most effective smoking cessation treatment available. Animal studies, especially in rats across laboratories and across specific vaccine compounds, have indicated that immunization leads to reduced nicotine penetration into the brain and diminished behavioral responses to nicotine, including locomotor effects [83,84]. However, the limited published human studies exploring various nicotine vaccines have not yet provided substantial evidence supporting vaccine efficacy as a treatment for nicotine dependence. To date, five nicotine vaccines have been tested across 16 Phase I–III clinical trials. However, due to insufficient anti-nicotine antibody production, insufficient specificity or affinity of antibodies produced from vaccination and high individual variability in antibody titer concentration post-vaccination, the expected results were not obtained [21,22,85]. Further research and clinical trials are needed to comprehensively assess the potential of vaccine-based treatments for nicotine dependence in humans.

### 5.3. Cocaine Vaccines

Cocaine, a highly addictive substance, necessitates innovative approaches for combating its abuse. Target based small-molecule approaches have not yielded highly effective medications to treat SUDs. Thus, pharmacotherapies, aimed to modulate or disrupt the drug’s effect at its site of action, have yet to be approved for the treatment of cocaine dependence. The direction of treatment shifts to the drug itself. Developing an active immunization against cocaine presents a potential solution by impeding the drug’s entry into the CNS, thereby obstructing its effects. To effectively intercept nearly all target drug molecules before they reach the blood–brain barrier, the antibody concentration in the bloodstream must be sufficiently high. Moreover, in order to maintain vaccine efficacy, it is crucial for this antibody level to remain consistent over a certain duration [7]. Importantly, such an approach is anticipated to yield fewer side effects compared to treatments focused on altering central neurotransmitter activity. 

The first reported anti-cocaine vaccine used unmodified cocaine as a hapten, conjugated to the surface of the cholera toxin B (CTB) subunit protein and co-administered with an alum adjuvant. It elicited a robust antibody response and hindered the pleasure associated with cocaine use [86]. Exploration of three rhesus monkeys immunized with a different cocaine vaccine (bovine serum albumin conjugate in alum) indicated that the immune response could induce a specific pharmacokinetic shift in cocaine distribution, effectively counteracting a behavioral response to the drug [87]. The route of administration may also be a factor affecting vaccine immunity. The presence of cocaine-specific antibodies in the mucosal lining after intranasal immunization might play a crucial role in impeding the direct entry of cocaine into the brain through the olfactory bulb. A cocaine vaccine combined with M7-NH2, a mucosal adjuvant and mast-cell-activating oligopeptide, could enhance cocaine-specific antibodies in mucosal secretions compared to the traditional adjuvant [88]. 

A drug-like hapten is another basic element used to design active vaccines. The cocaine hapten GNC was the initial subject of assessment, followed by GND, and more recently, GNE. These haptens could induce anti-cocaine antibodies with a strong affinity for binding free cocaine [89]. However, GNC-KLH was not able to overcome increasing doses or frequency of cocaine intake [90]. After achieving positive results in preclinical studies for GNC conjugated to CTB (TA-CD), this vaccine progressed to Phase II and Phase III clinical trials. Due to significant variability in antibody titers across different individuals, TA-CD has demonstrated constrained therapeutic effectiveness in promoting cocaine abstinence [25,26]. Subsequent research endeavors have aimed to enhance vaccine effectiveness through refining the hapten and adjuvant components. Three cocaine haptens containing fluorine (GNF, GNCF, and GN5F) and one containing chlorine (GNCl) were designed and synthesized, utilizing the chemical framework of succinyl norcocaine (SNC). Strategic incorporation of fluorine into haptens could enhance the efficacy of the existing cocaine vaccine in clinical trials and also introduce a promising novel strategy applicable to the development of vaccines targeting various substances of abuse [91]. A more recent investigation of a cocaine vaccine called dAd5GNE, which utilizes a third-generation cocaine hapten known as GNE (6-(2R,3S)-3-(benzoyloxy)-8-methyl-8-azabicyclo[3.2.1]octane-2-carboxamido-hexanoicacid) and is coupled to a modified serotype 5 adenovirus (Ad5) vector, indicated decreased cocaine levels in the brain. This vaccine also demonstrated a reduction in cocaine-induced hyperactivity and the motivation to self-administer cocaine [92,93,94,95]. dAd5GNE is another cocaine vaccine in addition to TA-CD that has progressed from preclinical to clinical trials. A Phase I clinical trial, aimed at evaluating the safety and immunogenicity of dAd5GNE, was commenced in 2015. However, as of now, no results from the trial have been made available in the published literature. The expected date of study completion is December 2025 [27]. Consequently, there always exists a pressing requirement to engineer vaccine formulations with enhanced immunogenicity, ultimately establishing vaccination as a viable therapeutic approach for addressing the challenges of cocaine abuse and addiction. A new vaccine candidate (UFMG-VAC-V4N2) demonstrated the production of elevated levels of anti-cocaine antibodies and exhibited a positive safety profile in a non-human primate model of Callithrix penicillate [28]. More recently, two innovative anti-cocaine immunogens based on calix[n]arene structures, designated as V4N2 and V8N2, promoted the production of cocaine antibodies and modulated the biodistribution of cocaine. At present, both V4N2 and V8N2 hold promise as potential candidates for the creation of immunogenic agents aimed at addressing cocaine use disorder [96]. However, more clinical trials are still needed to develop high-titer cocaine haptens and safe anti-cocaine vaccines.

### 5.4. Methamphetamine Vaccine

Methamphetamine (METH), an illicit psychostimulatory phenethylamine, induces a profoundly addictive and euphoric high by modulating monoamine neurotransmitter systems and increasing extracellular dopamine levels in the brain. Due to the involvement of multiple monoamine neurotransmitters, there are no established pharmacotherapies, namely receptor modulations, for the treatment of METH use disorder or overdose reversal at present. Therefore, researchers should utilize the field of immunopharmacotherapy with the aim of creating an active vaccine for METH. However, no MA vaccines have yet advanced to clinical trials.

Several preclinical studies have investigated the effectiveness of METH vaccines, yielding relatively promising outcomes. The initial active anti-METH vaccine did not produce a significant therapeutic effect. Then three haptenic compounds, MH2(R), MH6, and MH7, have been shown to produce antibodies with a high affinity and concentration capable of capturing the drug in the bloodstream [97]. Among these, MH6-KLH could block METH-induced locomotor and thermoregulatory disruptions in rats, exhibiting notable potential for developing a potentially significant METH vaccine [29]. Phenyl-substituted meth haptens have also been documented as active vaccination agents, such as SMO9. Repeated immunization with a high dose of SMO9-KLH did not result in any adverse effects on health, body weight, or performance during food-maintained behavioral testing. The antibodies generated by the SMO9-KLH vaccine were effective at sequestering METH in the bloodstream and prevented rats from METH-induced impairment of food responses [30]. Additional endeavors in the development of METH vaccines involve the utilization of a succinyl methamphetamine hapten (SMA)–KLH conjugate formulated with MPLA, which demonstrated a reduction in METH-induced locomotor effects in mice [98]. The SMA-TT vaccine attenuated acquisition and reinstatement of MA place conditioning. Furthermore, in vaccinated mice, the levels of METH in the brain were observed to decrease after the administration [31,99]. Despite the encouraging preliminary results, no methamphetamine vaccines have advanced to human clinical trials at this point.

## 6. Conclusions and Outlook

In comparison to the conventional pharmacological approach to treating SUDs via the administration of drug receptor agonists or antagonists, such as methadone, buprenorphine, or naltrexone, an immunotherapeutic strategy presents several inherent advantages as follows [100,101]:

1. Non-interaction with receptors in the CNS (do not act on the brain): The immunotherapeutic strategy has the potential to inhibit the drug’s activity without directly interacting with receptors (MORs, etc.). This approach does not elicit pharmacological effects that could potentially result in dependence or withdrawal. Moreover, these vaccines can be employed in conjunction with other medications, such as antidepressants and anti-craving agents;
2. Reduced side effects: Anti-drug antibodies do not directly engage with drug receptors in the brain or periphery. Consequently, the side effect profiles of these vaccines are significantly reduced compared to conventional pharmacological interventions, rendering them more tolerable for patients;
3. Long-lasting effects: The administered vaccine generates circulating antibodies that can remain in the body for up to a year, provided that strategically spaced booster injections are administered. This sustained effect offers extended protection against addiction and overdose risks, obviating the need for frequent dosing or patient compliance;
4. Reversible nature: the gradual attenuation of anti-drug antibodies over time confers on patients the capability to manage and potentially discontinue their vaccination regimen as necessitated.

Overall, anti-drug vaccines have the potential to effectively suppress addiction liability and reduce the risk of overdose associated with the targeted drug over a considerable period, providing a more manageable and patient-friendly treatment option. However, it is important to note that while the vaccine-generated IgG antibodies have long half-lives, and their duration of action is considerably longer than small molecules, they cannot ameliorate withdrawal symptoms [7,13,102]. Their primary function is to sequester the drug in the periphery and prevent it from crossing the BBB, thereby limiting the drug’s psychoactive effects [7]. As such, anti-drug vaccines serve as a promising approach to help address some aspects of drug abuse and overdose potential, but they may not address all aspects of SUDs treatment. As we all know, drug abuse immunotherapies are being developed primarily as therapeutic vaccines rather than prophylactic vaccines. Indeed, one of the potential risks associated with anti-drug vaccines is the possibility of drug overdose. This can occur if a patient, in an attempt to experience the rewarding effects of the drug, manages to override the effects of the anti-drug vaccines or change their method of drug intake (such as switching to injection or smoking). Such actions can reduce the amount of time available for anti-drug antibodies to bind to the free target drug, thereby potentially leading to significant harm or even fatal consequences for the patient [7,100,101]. Therefore, other therapeutic interventions, such as medications, monitoring, behavioral therapy, and peer support, may still be necessary to manage cravings and withdrawal symptoms effectively [4]. Potential downsides of anti-drug vaccines encompass the need for multiple injections and a time delay before a substantial immune response is achieved. Moreover, the extent of antibody response has exhibited some variability; that is, not all individuals attain sufficient antibody titers. These factors should be thoroughly evaluated in the ongoing research and development of anti-drug vaccines [103].

The question of whether immunizations can occur during drug administration is indeed crucial in the context of developing vaccine-based strategies for drug addiction and overdose prevention. To address this concern, two rodent studies have been conducted to evaluate vaccine efficacy under conditions of concurrent drug administration during the immunization process. The encouraging results from these studies have shown that there is no impairment of vaccine efficacy due to drug exposure. In other words, administering drugs alongside the vaccine during the immunization process does not hinder the generation of effective immune responses and the production of antibodies against the targeted drug. However, it is important to note that further research and clinical trials are necessary to validate these findings in humans and to assess potential interactions between drugs and vaccines in different scenarios. The safety and efficacy of immunizations during drug administration should be carefully evaluated to ensure that vaccine-based strategies are optimally effective in the context of SUDs.

Despite some challenges and initial failures, trials of anti-drug vaccines have indicated that they could be effective in combating SUDs if sufficient levels of titers are achieved in each patient. The success of these vaccines is highly dependent on proper vaccine design to generate antibodies with adequate concentration and strong affinity for the targeted drugs [34,103]. Recent advancements in anti-drug vaccine design have shown promising results, raising hopes that these vaccines may eventually prove successful in clinical settings. By improving various aspects of the design, such as hapten selection, carrier protein optimization, and adjuvant formulation, researchers have increased the likelihood of generating a robust and specific immune response against the drug of interest [42,43,104,105]. As with any medical development, further research and clinical trials will be crucial to establish the safety, efficacy, and applicability of drug conjugate vaccines as a viable strategy in the treatment of SUDs. The continuous progress in this area brings hope for more effective interventions in the future.

Certainly, while clinical results from anti-drug vaccines have not been entirely convincing, there are lessons that can be learned to improve their development. Here are some potential strategies to enhance the development of anti-drug vaccines:

- Focus on antibody function: Place greater emphasis on assessing the function of antibodies, particularly their affinity or avidity. The antigen’s structure, including the hapten, plays a crucial role in determining antibody function [42];
- Explore novel adjuvants: consider using new adjuvants that have shown promise in boosting both antibody quantity and affinity [104];
- Alternative administration routes: explore alternative administration methods beyond intramuscular injection, such as intranasal immunization, which could play a vital role in preventing cocaine’s direct entry into the brain through the olfactory bulb [106];
- Combination: Recognizing the multifaceted complexity of SUDs is essential, as they result from the interplay of various environmental and individual factors. This understanding implies that a multidisciplinary approach should be adopted in their treatment, involving the combination of vaccines with other addiction interventions (such as medications, monitoring, behavioral therapy, and peer support), or the use of multiple vaccines.
- The pursuit of effective anti-drug vaccines, although challenging, holds immense potential benefits, including the reduction of drug-related harm and enhancement of the quality of life for affected individuals. In the future, these vaccines may serve as a testament to the power of medical innovation in addressing one of the most pressing public health concerns of our time.

## Figures and Tables

**Table 1:** Summary of discussed anti-drug vaccines.

Drug	Clinical Trials	Animal Studies
Oxycodone/hydrocodone	Oxy(Gly)4-sKLH [11]	6OXY(Gly)4–KLH [12] OXY-dKLH [12] OXY-TT [13] Hydro-TT [13]
Fentanyl		fentanyl-TT [14] FEN-CRM + dmLT [15]
Heroin/morphine		M-TT [16] M-6-S-BSA [17] M-KLH [18] KLH-6-SM [19]
Nicotine	NicVAX [20] Niccine [21] Nic-Qb (NIC002) [22]	NanoNicVac [23] FH VLPs [24]
Cocaine	TA-CD [25,26] dAd5GNE [27]	UFMG-VAC-V4N2 [28]
Methamphetamine		MH6-KLH [29] SMO9-KLH [30] SMA-TT [31]
